# The association between amniotic fluid-derived inflammatory mediators and the risk of retinopathy of prematurity

**DOI:** 10.1097/MD.0000000000029368

**Published:** 2022-07-08

**Authors:** Ji Hye Jang, Jae-Gon Kim, Yu Hyun Lee, Jin Gon Bae, Jae Hyun Park

**Affiliations:** a Department of Ophthalmology, Keimyung University School of Medicine, Daegu, Republic of Korea; b Department of Obstetrics and Gynecology, Keimyung University School of Medicine, Daegu, Republic of Korea; c Department of Pediatrics, Keimyung University School of Medicine, Daegu, Republic of Korea.

**Keywords:** amniotic fluid, cesarean delivery, inflammatory mediators, retinopathy of prematurity, matrix metalloproteinase

## Abstract

Prenatal and perinatal infections and inflammation appear to associated with the development of retinopathy of prematurity (ROP). In this study, we evaluated whether inflammatory mediators in amniotic fluid (AF) retrieved during cesarean delivery influence the development of ROP in very low birth weight (VLBW) infants.

This retrospective study included 16 and 32 VLBW infants who did and did not develop any stage of ROP, respectively. Each infant with ROP was matched with 2 infants without ROP based on days of ventilation care, gestational age, and birth weight. AF was obtained during cesarean delivery, and the levels of intra-amniotic inflammatory mediators such as interleukin (IL)-1β, IL-2, IL-6, IL-8, IL-10, matrix metalloproteinase (MMP)-2, MMP-8, MMP-9, and tumor necrosis factor (TNF)-α were measured using a Human Magnetic Luminex assay (R&D Systems, Minneapolis, MN). The differences in the levels of inflammatory mediators according to the presence or absence of ROP were compared.

In patients who developed ROP, the level of MMP-2 in the AF was significantly increased (*P* = .011), whereas the levels of IL-10 and TNF-α were significantly decreased (*P* = .028 and .046, respectively) compared with those in infants who did not develop ROP. The levels of the other mediators were not significantly different between the 2 groups. Multivariate regression analysis showed that MMP-2 was a risk factor for the development of ROP (odds ratio, 2.445; 95% confidence interval, 1.170-5.106; *P* = .017).

The concentration of MMP-2 in AF is an independent factor in the development of ROP. Further studies are needed to determine whether the levels of inflammatory mediators in AF affect the ROP severity.

## 1. Introduction

Improvements in early prenatal and extremely preterm infant care have improved the survival rates of preterm babies. Retinopathy of prematurity (ROP) is a major cause of poor vision and blindness in survivors. ROP is a vasoproliferative disease of the retina that affects preterm infants and consists of 2 phases: (1) disruption of retinal vessel formation, and (2) abnormal retinal neovascularization.^[1,2]^ Angiogenesis is an important process in the development of ROP,^[1]^ involving various angiogenic and growth factors.^[3–5]^ In particular, it is well established that the interaction between vascular endothelial growth factor (VEGF) and insulin-like growth factor-1 is important in regulating retinal vascular development during the postnatal period.^[3–5]^

Gestational age (GA) and birth weight (BW) are the most powerful prenatal risk factors of ROP. However, numerous studies have focused on identifying antenatal risk factors. Some reports have proposed a multiphase ROP pathogenesis in which prenatal, perinatal, and postnatal inflammation contribute to the prophase of ROP, sensitizing the retina to ROP development.^[6,7]^ Although inflammatory mediators can modulate angiogenesis, their roles in ROP remain unclear. In a study investigating plasma cytokine levels in infants with early onset sepsis, interleukin (IL)-6, IL-8, and tumor necrosis factor (TNF)-α levels were associated with the development of ROP.^[8]^ In addition, several studies have shown that several inflammatory mediators in the cord blood of premature infants are associated with the occurrence of ROP.^[9,10]^ Moreover, other studies have shown that some inflammatory mediators in amniotic fluid (AF) and cord blood are higher or lower than normal in very low birth weight (VLBW) infants with bronchopulmonary dysplasia (BPD)^[11,12]^ or preeclampsia,^[13]^ which are associated with the development of ROP. Thus, prenatal and perinatal infections and inflammation, including chorioamnionitis, appear to be risk factors for ROP development.

Therefore, we hypothesized that ROP development may be related to intra-amniotic inflammatory mediators levels. In this study, we investigated the levels of inflammatory mediators in AF retrieved during cesarean delivery, and their potential usefulness as risk factors for ROP.

## 2. Patients and Methods

### 2.1. Ethical considerations

This observational cohort case-control study was conducted at the High-Risk Maternal and Newborn Integrated Care Center of the Keimyung University Dongsan Hospital (Daegu, Republic of Korea). This study was performed in accordance with the principles of the Declaration of Helsinki and approved by the Institutional Review Board of Keimyung University (approval number: 2021-07-049). Prior to AF collection, an agreement was signed with Keimyung Human Bio-Resource Bank, approved by the National Biobank of Korea. A.F. was provided by the Keimyung Human Bio-Resource Bank and had been approved by the Dongsan Medical Center Institutional Review Board (approval number: 2020-01-001). The authors obtained written consent from the parents for A.F. collection and the use of data for research purposes.

### 2.2. Study design and setting

**Figure 1.**
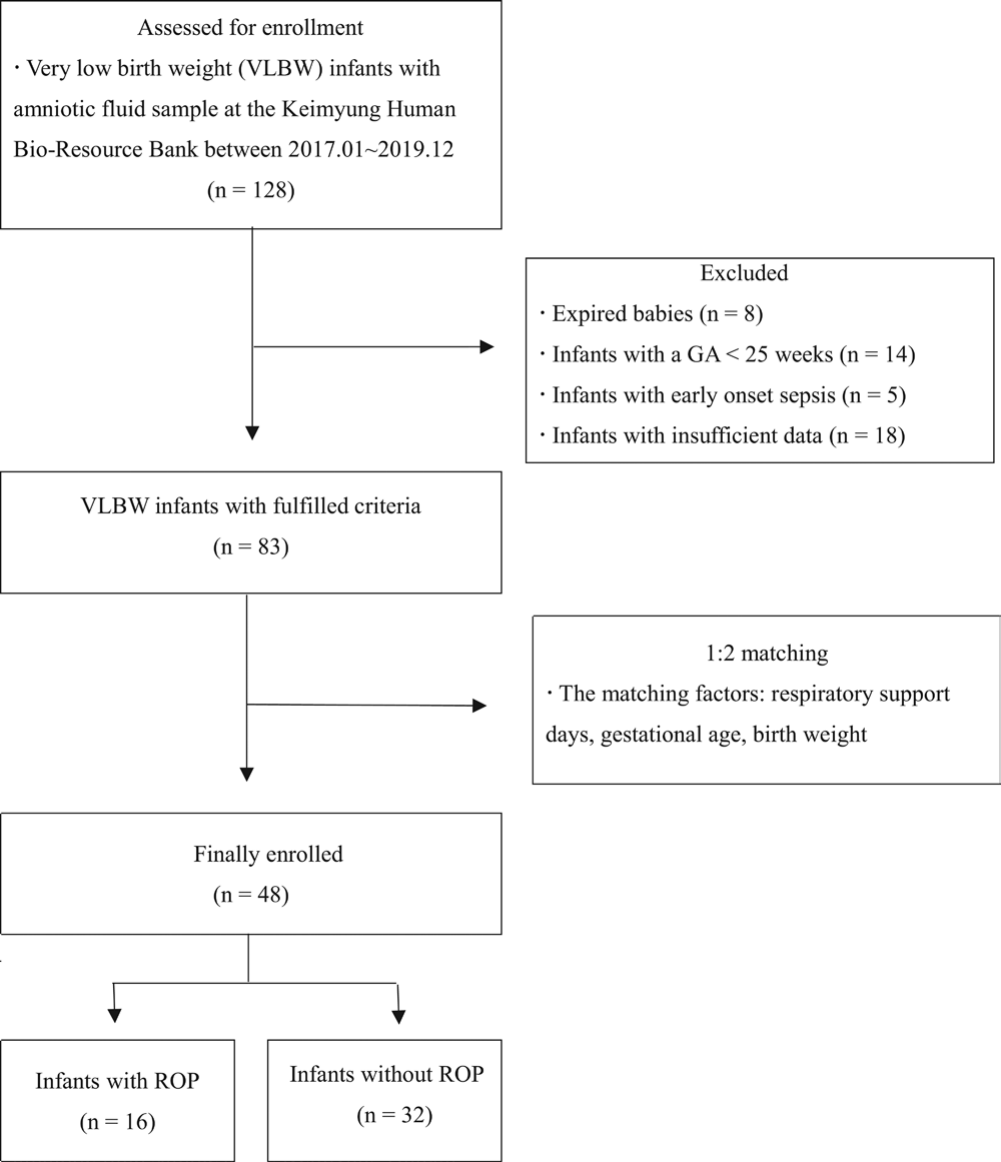
Flow diagram illustrating the enrollment process.

We investigated VLBW infants who were born weighing <1500 g with AF stored at the Keimyung Human Bio-Resource Bank between January 2017 and December 2019. We excluded more immature preterm infants with GA < 25 weeks and those with early onset sepsis. We also excluded preterm infants who died before the first ocular examination or during follow-up examinations and those who were lost to follow-up. A 1:2 case-control match design was used to reduce bias.^[14]^ According to the number of respiratory support days, each infant with ROP was matched with 2 infants without ROP. In the case of 3 or more control groups with the same number of days of ventilation care, the infants with the most similar GA were matched, followed by the infants with the most similar BW. A total of 48 infants (32 without ROP and 16 with ROP) were enrolled in this study (Fig. 1).

### 2.3. Maternal and neonatal clinical data

Maternal characteristics included maternal age, parity, multiple pregnancies, cerclage intervention, preterm labor, preterm premature rupture of membrane (PPROM), placenta previa, placental abruption, preeclampsia, oligohydramnios, intrauterine growth retardation, and histological chorioamnionitis. GA was estimated based on the last menstrual period of the mother and the ultrasonography findings. Cerclage intervention was defined as prophylactic operative intervention for the treatment of painless cervical dilation in the second trimester. Preterm labor was defined as regular contractions of the uterus resulting in changes in the cervix. PPROM was defined as membrane rupture within 24 hours before the onset of labor. The presence of inflammation in placental tissue was indicative of histological chorioamnionitis.

The neonatal characteristics included GA at birth, BW, Apgar scores (1 and 5 minutes), ventilator support days, nasal respiratory support days, and BPD. BPD was defined as chronic lung disease that required supplemental oxygen support for at least 28 days.

### 2.4. Ocular examination

The first ocular examination of all preterm babies was performed at 31 weeks postmenstrual age or 4 to 6 weeks postnatal age in the neonatal intensive care unit according to the ROP screening guidelines.^[15]^ The fundus was examined using an indirect ophthalmoscope with a 20 D lens under topical anesthesia after pupil dilatation. Retinal images were obtained using a wide-angle 130° retinal camera (RetCam; Clarity Inc., Pleasanton, CA, USA). The classification of ROP was based on the guidelines established by the Early Treatment for Retinopathy of Prematurity Cooperative Group^[16]^ and the revised International Classification of Retinopathy of Prematurity.^[17]^

Follow-up examinations are recommended according to the state of the retinal vascular development. Eyes that did not develop ROP were followed up until full vascularization was observed in the peripheral retina. Eyes with mild ROP (stage 1 or 2) were followed up until complete regression. Eyes with severe ROP (stage 3 or 4A) that required treatment received an intravitreal injection of an anti-VEGF agent (bevacizumab, Avastin; Genentech, Inc., San Francisco, CA).

### 2.5. Inflammatory mediators in the AF

After obtaining informed consent from pregnant women at risk of preterm birth at 24 to 34 weeks GA, we collected AF from the amniotic sac during cesarean delivery. Amniocentesis was not performed in patients under general anesthesia, severe oligohydramnios (AF index < 1.0), massive vaginal bleeding, or unexpected amniotic sac rupture to reduce the risk in pregnant women and infants during labor. The collected AF was centrifuged, and the supernatant was aliquoted and stored at –80°C at the Keimyung Human Bio-Resource Bank. The samples were not subjected to freeze-thaw cycles before being assayed. For protein analysis, the samples were thawed and centrifuged at 15,000 rpm for 10 minutes at 4°C. The supernatants from the samples were used to measure the levels of inflammatory mediators. Intra-amniotic inflammation engenders a maternal and fetal inflammatory response characterized by the release of inflammatory mediators such as IL-1β, IL-2, IL-6, IL-8, IL-10, TNF-α, MMP-2, MMP-8, and MMP-9.^[18]^ Nine inflammatory mediators of the AF were quantitatively measured using a Human Magnetic Luminex screening assay (R&D Systems, Minneapolis, MN) on a Bio-Plex 200 (Bio-Rad, CA). All the measurements were performed strictly according to the manufacturer’s instructions and all samples were measured in duplicate at the same time. For the IL-1β assay, a standard curve was developed from 39.0 to 9488.0 pg/mL with a sensitivity of 1.6 pg/mL; for the IL-2 assay, the curve was linear from 59.2 to 14,400.0 pg/mL with a sensitivity of 3.6 pg/mL; for the IL-6 assay, the curve was linear from 9.6 to 2308.0 pg/mL with a sensitivity of 3.4 pg/mL; for the IL-8 assay, the curve was linear from 10.4 to 2510.0 pg/mL with a sensitivity of 3.6 pg/mL; for the IL-10 assay, the curve was linear from 9.6 to 2324.0 pg/mL with a sensitivity of 3.2 pg/mL; for the TNF-α assay, the curve was linear from 19.4 to 4718.0 pg/mL with a sensitivity of 2.4 pg/mL; for the MMP-2 assay, the curve was linear from 16,640.0 to 3921,650.0 pg/mL with a sensitivity of 5400.0 pg/mL; for the MMP-8 assay, the curve was linear from 490.2 to 119,124.0 pg/mL with a sensitivity of 68.4 pg/mL; and for the MMP-9 assay, the curve was linear from 6705.0 to 1629,800.0 pg/mL with a sensitivity of 680.0 pg/mL.

### 2.6. Data analysis

Statistical analyses were performed using IBM SPSS Statistics 25.0 (IBM Co., Armonk, NY). Statistical significance was set at *P* values < .05.

Maternal and neonatal characteristics were analyzed using the Mann–Whitney *U* test (for continuous variables) or Fisher exact test (for categorical variables), respectively. Prior to statistical analysis, the values of the levels of inflammatory mediators were log2 transformed. The mean levels of inflammatory mediators in the AF were compared according to the presence or absence of ROP using the Mann–Whitney *U* test. Multivariate logistic regression analysis adjusted for covariates (e.g., GA, ventilator support days, and nasal respiratory support days) was used to estimate the odds ratio with a 95% confidence interval to identify the relationship between inflammatory mediators in AF and the development of ROP.

## 3. Results

Among the 16 VLBW infants in the ROP group, 11 (69%) had stage 1 ROP, 3 (19%) had stage 2 ROP, and 2 (12%) had stage 3 ROP. Infants with stage 3 ROP received an intravitreal injection of bevacizumab (0.625 mg in 0.025 ml, half the dose in adults) in both eyes. None of the infants had stage 4 ROP or higher disease.

Table 1 shows maternal and neonatal characteristics according to the presence or absence of ROP. There were no statistically significant differences in demographic characteristics, including preterm labor, PPROM, and histological chorioamnionitis. The GA and BW of VLBW infants in the ROP group were lower than those in the non-ROP group. Nasal respiratory support days were higher in infants with ROP than in those without ROP. Consequently, BPD rate was higher in infants with ROP than in those without ROP. However, these differences were not statistically significant.

**Table 1:** Maternal and neonatal characteristics according to the presence or absence of retinopathy of prematurity in very preterm infants

Parameters	Control group (n = 32)	ROP group (n = 16)	*P*
	33 ± 4	33 ± 6	.683
Multiple pregnancy, n (%)	14 (43.8)	7 (43.8)	1.00
Cerclage intervention, n (%)	6 (18.8)	1 (6.3)	.398
Preterm labor, n (%)	23 (71.9)	9 (56.3)	.339
Preterm premature rupture of membrane, n (%)	9 (28.1)	3 (18.8)	.725
Placenta previa, n (%)	3 (9.4)	0 (0.0)	.541
Placenta abruption, n (%)	1 (3.1)	1 (6.3)	1.00
Preeclampsia, n (%)	10 (31.3)	7 (43.8)	.524
Gestational diabetes, n (%)	6 (18.8)	2 (12.5)	.701
Oligohydramnios, n (%)	6 (18.8)	2 (12.5)	.701
Intrauterine growth retardation, n (%)	5 (15.6)	1 (6.3)	.648
Histologic chorioamnionitis, n (%)	4 (25)	16 (50)	.127
Gestational age at birth (weeks)	30.0 ± 1.3	29.2 ± 1.6	.104
Weight at birth (g)	1343 ± 291	1294 ± 318	.614
Apgar 1 min score	6.0 ± 1.3	6.3 ± 1.0	.501
Apgar 5 min score	7.8 ± 0.8	7.8 ± 0.8	.962
Respiratory distress syndrome, n (%)	25 (78.1)	14 (87.5)	.697
Ventilator support (days)	5.7 ± 7.8	5.8 ± 8.1	.990
Nasal respiratory support (days)	19.6 ± 18.4	30.1 ± 23.9	.135
Bronchopulmonary dysplasia, n (%)	10 (31.25)	9 (56.25)	.124

*P* values were calculated using the Mann-Whitney *U* test and Fisher exact test.

n = number, ROP = retinopathy of prematurity, SD = standard deviation.

The levels of inflammatory mediators in the AF were compared according to the presence or absence of ROP (Table 2). The concentration of MMP-2 in the ROP group was significantly higher than that measured in the group without ROP (18.4 ± 0.8 vs 17.6 ± 1.0, respectively, *P* = .011). In contrast, the levels of IL-1β, IL-10, TNF-α, and MMP-9 were significantly lower in the ROP group than in the non-ROP group (*P* < .05).

**Table 2:** Comparison of inflammatory mediators in amniotic fluid retrieved during cesarean delivery according to the presence or absence of retinopathy of prematurity

Parameters	Control group (n = 32)	ROP group (n = 16)	*P*
MMP-2	17.6 ± 1.0	18.4 ± 0.8	.011*
MMP-8	13.0 ± 3.8	12.3 ± 2.8	.487
MMP-9	11.8 ± 3.8	10. 1 ± 1.7	.034
IL-1β	5.1 ± 2.0	5.5 ± 2.9	.015
IL-2	4.7 ± 2.2	4.3 ± 1.9	.571
IL-6	11.7 ± 5.1	10.0 ± 3.2	.175
IL-8	10.9 ± 2.2	10.5 ± 1.8	.609
IL-10	4.1 ± 2.5	2.8 ± 1.2	.016*
TNF-α	5.3 ± 3.6	3.4 ± 1.3	.011*

Values are presented as the mean ± standard deviation.

* Statistically significant (*P* < .05).

IL = interleukin, MMP = matrix metalloproteinase, ROP = retinopathy of prematurity, TNF = tumor necrosis factor.

The results of the multivariate logistic regression analysis are presented in Table 3. The level of MMP-2 in AF was significantly associated with the development of ROP under the control of GA, ventilator support days, and nasal respiratory days (odds ratio, 2.445; 95% confidence interval, 1.170-5.106; *P* = .017).

**Table 3:** Multivariate logistic regression analysis associated with development of retinopathy of prematurity

Parameters	Adjusted OR	95% CI	*P*
MMP-2	2.445	1.170-5.106	.017*
MMP-9			.193
IL-1β			.131
IL-10			.168
TNF-α			.110
Gestational age			.067
Ventilator support			.965
Nasal respiratory support			.096

* Statistically significant (*P* <.05).

CI = confidence interval, IL = interleukin, MMP = matrix metalloproteinase, OR = odds ratio, TNF = tumor necrosis factor.

## 4. Discussion

We investigated the relationship between inflammatory mediators in AF and development of ROP in VLBW infants. The major finding of our study was that levels MMP-2 in AF contributed to the risk of ROP. In addition, the differences between this study and other studies are as follows: (1) AF was obtained through cesarean delivery and (2) the use of ventilation days, GA, and BW were controlled between the groups with and without ROP during the analysis.

In our study, the AF was retrieved during cesarean delivery. In previous studies, the AF was collected using an ultrasound-guided transabdominal approach before delivery.^[13,19]^ This procedure could be accompanied by rare complications such as preterm labor, placental abruption, PPROM, and fetal heart rate abnormality.^[20]^ In particular, emergent cesarean delivery after transabdominal amniocentesis is a major complication that should be considered during the third trimester.^[21,22]^ Therefore, a notable difference in our study was that all the AF collected during preterm delivery at 24 to 34 weeks of gestation was obtained during cesarean delivery. AF can be obtained by puncture under direct visualization after uterine incision during cesarean delivery without the risk of such complications. In addition, investigation of AF collected during cesarean delivery seems to adequately reflect the state of intra-amniotic inflammation at the time of delivery.

In our study, patients were matched in a 1:2 ratio in the groups with and without ROP according to mechanical ventilator days, GA, and BW. The major risk factors for ROP are GA and BW at birth. Also, there is no doubt that prolonged ventilator use and increased oxygen demand are the main causes of ROP. Therefore, it is essential to interpret the relationship between intra-amniotic inflammation and ROP development after excluding more immature infants with a GA < 25 weeks.

A recent study showed a relationship between inflammatory and angiogenic mediators in AF and ROP development.^[19]^ AF was obtained from 175 premature infants born between 23 and 32 weeks gestation. Culture tests were performed and the concentrations of various mediators were measured. This study revealed that elevated levels of IL-6, IL-8, endoglin, and insulin-like growth factor-binding protein 2 in AF were independently associated with the occurrence and progression of ROP in preterm infants. However, the study did not control for ventilation care or GA, which are strong risk factors of ROP. In our study, mechanical ventilator days, GA, and BW were controlled by dividing the patients into groups with and without ROP. In addition, the multivariate logistic regression analysis adjusted for GA, ventilator support days, and nasal respiratory support days showed no significant differences according to the presence or absence of ROP.

In terms of the concentration of inflammatory mediators in the AF, MMP-2 levels were high in the ROP group, whereas IL-10 and TNF-α levels were low. Multivariate regression analysis revealed that MMP-2 is a significant risk factor for ROP.

MMPs, a family of proteinases that digest the extracellular matrix, play important roles in tissue remodeling processes such as angiogenesis, tissue repair, and cancer metastasis.^[23–26]^ MMP-2 destroys type IV collagen (a component of the basement membrane) and initiates angiogenesis together with membrane-type MMPs and MMP-9.^[27,28]^ Moreover, MMP-2 is associated with pathologic retinal neovascularization.^[29,30]^ If a fetus exposed to high MMP-2 in AF is born prematurely, the retina may be sensitive and may be involved in the formation of abnormal new blood vessels in the immature retina. In this study, we found significantly higher MMP-2 levels in the AF of the ROP group than in that of the control group.

IL-10 exerts important anti-inflammatory effects by suppressing the immune response, acting as a negative regulator of the local cytokine microenvironment, and limiting antigen presentation.^[31]^ The lack of IL-10 enhances T-cell activation and differentiation, resulting in a strong inflammatory reaction to infection.^[32]^ TNF-α, a potent pro-inflammatory cytokine produced by macrophages and monocytes, has paradoxical anti-inflammatory properties.^[33–36]^ In this study, significantly lower IL-10 and TNF-α levels were observed in the ROP group than in the non-ROP group. Low IL-10 and TNF-α concentrations in AF may sensitize the immature retina during the fetal period, thereby contributing to a predisposition to ROP. Some investigations have yielded results inconsistent with those obtained in the present study regarding the relationship between IL-10 and TNF-α cytokines and ROP. Woo et al^[10]^ showed that the levels of IL-10 and TNF-α in umbilical cord blood did not differ between ROP and control groups. Other studies have shown that high concentrations of TNF-α are associated with ROP development.^[8,37]^ These different results may be attributed to the different locations and times at which samples were collected.

The present study has some limitations. First, although this was the first ventilation-care-matched case-control study, it had a retrospective design. Therefore, the possibility of a selection bias in infant inclusion and matching cannot be completely excluded. Second, the study involved a relatively small number of cases and was conducted at a single center. Therefore, it may be difficult to apply these results to an entire population. Third, we excluded more immature preterm infants with a GA < 25 weeks and those exposed to early onset sepsis, which may have biased the elucidation of the relationship between intra-amniotic inflammation and ROP. A prospective study investigating the relationship between intra-amniotic inflammation and ROP, including more immature infants with various morbidities, is needed. Fourth, various angiogenic and growth factors associated with ROP were excluded from this study. A comparison of the relationship between inflammatory cytokines and angiogenic or growth factors in AF is necessary for the prenatal investigation of the occurrence of ROP. Fifth, duplicate measurement using the enzyme-linked immunosorbent assay method for quantitative analysis of inflammatory mediators in AF cannot statistically exclude outliers. More repeatable measurements may be helpful in delicate analyses. Finally, since only 3 cases with severe ROP stage > 3 were included in this analysis, it was not possible to confirm the effect of intra-amniotic cytokines on the severity of ROP. Therefore, a prospective follow-up study with a larger number of children and infants with severe ROP is required. Nonetheless, this is the first study to assess the relationship between the levels of inflammatory mediators in AF and postnatal development of ROP under ventilation control.

## 5. Conclusion

In conclusion, when ventilation support days were matched in VLBW infants, the concentration of MMP-2 in the AF was an independent factor for the development of ROP. Further studies are needed to determine whether the levels of inflammatory cytokines in AF affect ROP severity. This approach may assist in confirming the possibility of ROP development through prenatal evaluation of AF in infants who are likely to be born prematurely, thereby enabling physicians to warn caregivers.

## Acknowledgments

The specimens used in this study were provided by the Biobank of Keimyung University Dongsan Hospital Biobank, which is a member of the Korea Biobank Network.

## Author contributions

Study concept and design: Ji Hye Jang, Jae Hyun Park

Collection of amniotic fluid: Jin Gon Bae

Molecular biology analysis based on the amniotic fluid: Jae Hyun Park

Neonatal ocular examination: Ji Hye Jang

Acquisition and analysis of data: Ji Hye Jang, Jae-Gon Kim, Yu Hyun Lee and Jae Hyun Park

Writing of the article: Ji Hye Jang, Jae-Gon Kim, Jae Hyun Park

Revision of the article, Study supervision: All authors
